# ^230^Th dating of flowstone from Ignatievskaya Cave, Russia: Age constraints of rock art and paleoclimate inferences

**DOI:** 10.1002/gea.21851

**Published:** 2021-02-24

**Authors:** Yuri Dublyansky, Vladimir Shirokov, Gina E. Moseley, Pavel A. Kosintsev, R. Lawrence Edwards, Christoph Spötl

**Affiliations:** ^1^ Institute of Geology Innsbruck University Innsbruck Austria; ^2^ Institute of History and Archeology, Ural Branch Russian Academy of Sciences Yekaterinburg Russian Federation; ^3^ Institute of Plant and Animal Ecology, Ural Branch Russian Academy of Sciences Yekaterinburg Russian Federation; ^4^ Department of Earth Sciences University of Minnesota Minneapolis Minnesota USA

**Keywords:** ^230^Th dating, Paleolithic cave art, permafrost, Ural

## Abstract

Paleolithic antiquity of parietal art in Ignatievskaya cave, Southern Ural, is supported by its subject (Late Pleistocene animals) as well as by paleontological and palynological data, and ^14^C dates from cultural layers associated with artistic activity (17.8−16.3 cal ka BP; association is established by finds of ochre in these layers). However, three ^14^C dates of charcoal motifs yielded younger, Holocene ages (7.4−6.0 cal ka BP). In this study, we constrain the age of parietal art in the cave by ^230^Th dating of flowstone that brackets the paintings. Flowstone did not form in the cave between c. 78 and 10 ka BP, due to widespread permafrost in northern Eurasia at that time. Our ^230^Th dates do not support the middle Holocene age of art in Ignatievskaya cave and are consistent with its Upper Paleolithic antiquity instead.

## Graphical abstract

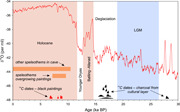

## 1 INTRODUCTION

The caves Kapova (Shulgan‐Tash) and Ignatievskaya (Yamazy‐Tash) host the easternmost occurrences of Paleolithic cave art in Europe. The caves are located in the Southern Urals, Russia, more than 4,000 km east of their Western European counterparts.

Parietal art in the Shulgan‐Tash cave was discovered earlier (1959) than in Ignatievskaya (1980), and it has, therefore, been studied in somewhat greater detail. The Upper Paleolithic age of paintings in Shulgan‐Tash is firmly established on the basis of several independent lines of evidence. Paintings feature naturalistic representations of the Pleistocene megafauna: mammoth, woolly rhinoceros, horse, bison, and Bactrian camel. Noting the stylistic similarities with the Franco‐Cantabrian art, Bader (1965) posited that paintings in Shulgan‐Tash are equivalent to the late Solutrean–mid‐Magdalenian cultures of Western Europe. Subsequent ^14^C dating of charcoal and bones from cultural layers in the cave proved that this attribution was accurate (Kotov, 2014; Šcelinskij & Širokov, 1999; Zhitenev et al., 2015). The ^14^C dates from cultural layers in Shulgan‐Tash are relevant to cave‐art studies due to the discovery of ochre “crayons” and painted fragments of rock and bones within these layers (Šcelinskij & Širokov, 1999). Such findings unequivocally link the cultural layer with the artistic activity in the cave. The Upper Paleolithic age of the paintings in Shulgan‐Tash was further supported by ^230^Th dating of flowstone on which some paintings were made and by which some were overgrown (Dublyansky et al., 2018).

Similar to Shulgan‐Tash, figurative images in Ignatievskaya cave feature members of the Pleistocene megafauna including mammoths and a rhinoceros‐like animal. Artifacts of the Paleolithic and the Bronze and Iron Ages were found in the cave, of which only Paleolithic materials were associated with a clearly identifiable, albeit thin, cultural layer; younger finds were collected either from the cave floor or found in loose sediments (Petrin, 1992; Shirokov & Petrin, 2013). The cultural layer also contained abundant fragments of charcoal and rare clumps of ochre, which establishes a link between this layer and the red paintings (Shirokov & Petrin, 2013). Three early conventional ^14^C dates of charcoal and bones from the cultural layer yielded Upper Paleolithic ages (18.3–15.4 cal ka BP; i.e., thousands of years [ka] before 1950 C.E.; common era; Petrin, 1992). The chronology was recently refined by accelerator mass spectrometry (AMS) dating of charcoal, with four dates constraining the time of its accumulation to 17.4–16.3 cal ka BP and one sample (from the deepest position) yielding an older age of 28.3–27.8 cal ka BP (Dublyansky et al., 2021; see Supporting Information S4). The dates are consistent with paleontological evidence from the cultural layer, represented by Late Pleistocene fauna (Petrin, 1992) and pollen characteristic of uniform, forest‐free vegetation of a periglacial steppe (Smirnov et al., 1990).

Although these lines of evidence, as well as the similarity with the Shulgan‐Tash cave, make a seemingly strong case for the Upper Paleolithic antiquity of the parietal art in Ignatievskaya cave, some ambiguity remains. First, the similarity between these two decorated Southern Ural caves is limited, due to the abundance of black (charcoal) paintings in Ignatievskaya cave, which are all but absent in Shulgan‐Tash. Second, stylistically, figurative paintings in the two caves are quite distinct. Some researchers suggested that styles of figurative paintings in Ignatievskaya cave are not characteristic of the Paleolithic “naturalism” (Formozov, 1998; 2000; Zhitenev, 2016). Others countered that the “Ignatievskaya” style is not consistent with any post‐Paleolithic art known in Southern Ural either (Shirokov, 2006). Third, three ^14^C determinations made directly on the charcoal paintings returned Mesolithic rather than Upper Paleolithic dates (8.9–6.6 cal ka BP; Shirokov et al., 2003; Steelman et al., 2002).

In this study, we provide independent constraints on the age of paintings in Ignatievskaya cave employing ^230^Th dating of flowstone that formed before and after the pigment was applied. This approach has recently established itself as a valuable and robust tool in the field of cave art dating (Hellstrom, 2012; Hoffmann et al., 2016; 2018; Pike et al., 2012; Pike, 2017).

## 2 MATERIALS AND METHODS

### 2.1 Materials

In this paper, we present results of the geochronological study of eight samples of speleothems (flowstone and a stalagmite) from Ignatievskaya cave. Five samples have identifiable temporal relationships with parietal art, whereas another three were used to build confidence in ^230^Th dating results and characterize the speleothem/paleoclimate system.

### 2.2 Sampling

Samples of flowstone were collected as 8‐mm‐diameter cores using hand‐held drilling equipment (Spötl & Mattey, 2012). In the laboratory, the cores were cut longitudinally with a precision diamond saw (Buehler ISOMET). Cut surfaces were polished to better reveal growth zoning and enable precise milling of subsamples. Subsamples for ^230^Th dating (6–200 mg) were milled in the laboratory in a clean air laminar‐flow hood.

### 2.3 ^230^Th dating

Samples were spiked with a mixed ^229^Th–^233^U–^236^U tracer before chemical separation of U and Th using an Fe coprecipitation procedure, followed by chemical purification on anion exchange columns (Edwards et al., 1987; Shen et al., 2012). Procedural chemistry blanks were typically less than 100 ag for ^230^Th and less than 1 fg for ^234^U. U and Th isotopic ratios and concentrations were determined at the University of Minnesota, MN, using the latest protocols on a Thermo Finnigan Neptune Multi Collector Inductively Coupled Plasma Mass Spectrometer (Shen et al., 2012) and the half‐lives of Jaffey et al. (1971) and Cheng et al. (2013). In line with standard practice, age corrections assume an initial ^230^Th/^232^Th atomic ratio of 4.4 ± 2.2 × 10^−6^ of bulk Earth (Wedepohl, 1995).

### 2.4 ^230^Th age modeling

For specimens with multiple ^230^Th dates, age modeling was performed using OxCal 4.4 (Bronk Ramsey, 2008; Bronk Ramsey & Lee, 2013) to assess the time of the initiation of speleothem growth.

### 2.5 Calibration of ^14^C ages

All radiocarbon ages referred to in this paper were taken from publications. For consistency, the original ^14^C ages were recalibrated using the program OxCal 4.4 (Bronk Ramsey, 2008) and the IntCal20.14c calibration curve (Reimerr et al., 2020).

## 3 IGNATIEVSKAYA CAVE

**Figure 1:**
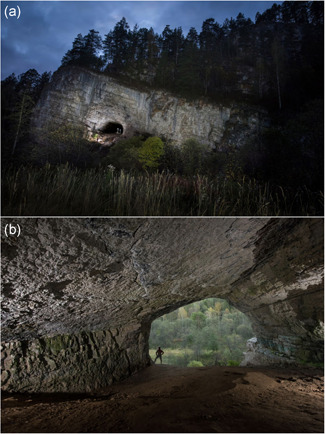
Entrance to Ignatievskaya cave: (a) Cave opens up in a limestone cliff 12 m above the Sim river; (b) The entrance gallery looking outside. The bedding plane of the bedrock is dipping 5°–20° to the left. *Photos*: R. Shone [Color figure can be viewed at wileyonlinelibrary.com]

**Figure 2:**
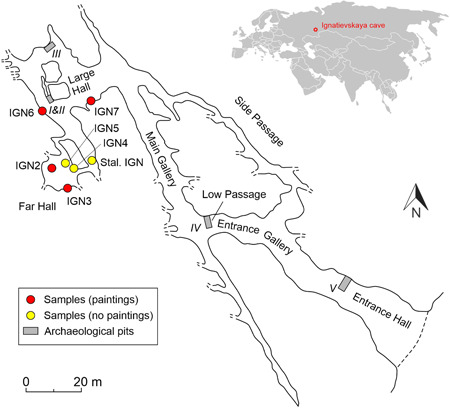
A plan view of Ignatievskaya cave, showing positions of samples and location of archaeological pits, from which ^14^C dates were obtained (Smirnov et al., 1990; Petrin, 1992; Dublyansky et al., 2021). The insert shows location of the Ignatievskaya cave in Eurasia [Color figure can be viewed at wileyonlinelibrary.com]

Ignatievskaya Cave (54°53ʹN, 57°46ʹE) is located on the western slope of the Ural Mountains. The portal of the cave, some 12 m in diameter, opens up in a 70‐m‐high cliff of Devonian limestone, 12 m above the Sim river (Figure 1). The 800‐m‐long cave is nearly horizontal. It consists of a series of long, NW‐ and NNW‐oriented galleries, connected by shorter E‐ and ESE‐oriented passages (Figure 2). Most of the cave passages are spacious, with an average height of c. 2.5 m and a width of c. 3 m. Only access to Far Hall requires crawling through narrow, tube‐shaped passages. In the first half of the 19th century, the Far Hall served as a hermitage for Orthodox monk Ignatiy (hence the Russian name of the cave). Ignatiy's secluded life was revered by the local population, and after his death, on major church holidays, the cave was crowded with people (Chernyshov, 1886). Abundant use of open fire (torches) at that time resulted in blackening most of the interior cave walls with soot.

The Entrance Hall and the Entrance Gallery are spacious and open. The temperature (*T*) and the relative humidity (*RH*) in this part of the cave change in concert with the outside conditions; however, the changes become progressively attenuated as the distance from the cave entrance increases. The interior of the cave, beyond the Low Passage, has a stable microclimate (Large Hall: *T* = 5.1 ± 0.1°C, *RH* ~98%; Shirokov & Petrin, 2013).

The first archaeological excavations were made in the cave in early 20th century. Only materials dating back to the Bronze Age and Medieval Period (ceramics; iron arrowhead) were found at that time (Rudenko, 1914). In 1960–1961, in addition to the Holocene‐age artifacts, five Upper Paleolithic stone tools were found in archaeological pits dug in the near‐entrance part of the cave (Bader, 1980). In 1980, archaeologists V. Petrin, S. Chairkin, and V. Shirokov discovered Paleolithic parietal art in the cave. During a comprehensive 6‐year‐long cave research program that followed, five pits were excavated in different parts of the cave (Figure 2). The thin Paleolithic cultural layer (“visiting horizon”) was identified at a depth of 0.05 to 0.25 m in pits I−IV, excavated in the deep aphotic part of the cave (Petrin, 1992). In addition to stone and bone tools and decorations made of bone, the layer contained abundant small fragments of charcoal and ochre as well as animal bones (Shirokov & Petrin, 2013). Archaeological finds were scarce in the interior of the cave (only 28 artifacts were found in the Large Hall) but abundant in the Entrance Hall and the Low Passage (more than 1,300 artifacts).

The paintings are located c. 120 m from the cave entrance, in the Large and Far Halls (Petrin, 1992; Šcelinskij & Širokov, 1999). Today's inventory comprises about 180 locations with paintings or their fragments (Shirokov & Petrin, 2013). Both red (ochre) and black (charcoal) paintings are present. The motifs can be subdivided into nonfigurative (lines, dots, etc.) and figurative ones, among which both realistic (mammoth, horse) and composite and nonrealistic zoomorphic figures are known.

The question of the temporal relationships between the black and red paintings in the cave remains moot. At one location, in the Large Hall, a red line was found to be drawn across a black line, indicating that this particular black motif is older than the red one; however, it remains unknown how much older it is and whether this applies to other black and red paintings in the cave (Shirokov & Petrin, 2013).

## 4 SPELEOTHEMS IN IGNATIEVSKAYA CAVE

*Speleothem* is a collective term describing mineral deposits formed in caves by chemical precipitation from flowing, dripping, ponded, or seeping water (White, 2019). Compared with many of its Franco‐Cantabrian counterparts, Ignatievskaya cave is poorly decorated with speleothems; most of the halls and passages of the cave are devoid of them.

The term *flowstone* refers to one of the common types of speleothems, forming via precipitation of calcite from a thin film of water flowing along inclined surfaces (cave walls, ceilings, and floors). Typically, flowstone has a sheet‐like appearance (i.e., width and length of the deposit are much greater than its thickness) and consists of superimposed layers of minute calcite crystals growing with their c‐axes perpendicular to the sheet (White, 2019).

Many flowstone occurrences in Ignatievskaya cave (some of which have been sampled in this study) have a somewhat less common “runnel” morphology (Figures S1–1). Water, from which the flowstone was deposited, did not wet the entire surface. Rather, it formed 1 to 2‐cm‐wide runnels, whose lengths range from tens of centimeters to several meters. Deposition of calcite was largely restricted to these runnels. Initial layers were deposited as sub‐mm‐thin lamina. As precipitation continued, small calcite “trails” formed on inclined surfaces of cave walls began to channel the flow. New calcite layers enveloped previously formed ones, resulting in a gradual thickening of the calcite, eventually forming inverted ridge‐shaped deposits, whose length is significantly greater than their width and thickness. The height of the ridges varies from a few to 10−15 mm. The cave wall on the sides of such ridges is either barren or coated with a sub‐mm‐thin flowstone.

### 4.1 Sampling strategy

The sampling strategy was specifically designed with conservation in mind keeping damage and disturbance to a minimum. Places where paintings and speleothems occur together are not common in the cave, and those parts of the cave where such relationships can be observed are relatively small. Accordingly, we started with a thorough examination of all locations where spatiotemporal relationships between flowstone and paintings could be assessed.

Sampling for ^230^Th dating involved drilling seven mini‐cores (8 mm in diameter) from the flowstone. To avoid damage to paintings underlying the flowstone, in all but one case, our cores were taken outside of, but fairly close to, the painted areas. The obvious downside to this approach is that the paint layer is only visible in one of our cores, but in the interest of preservation, we deemed this absolutely necessary. The spatiotemporal relationships between paintings and flowstone, therefore, needed to be ascertained by other means, such as detailed observations on the flowstones and paintings.

Our observations in the cave confirm that speleothems are not abundant and, in many cases, exhibit clearly identifiable functional relationships. For example, we observed that small stalagmites on the floor of the Far Hall are part of the same speleothem‐forming system as runnel flowstones on its ceiling (Figures S1–2). This field determination was subsequently confirmed by ^230^Th dating. Stalagmites are thicker and allow fine‐scale sampling as compared with thin runnel flowstones overgrowing paintings. These speleothems were sampled to provide more a robust reconstruction of the speleothem growth history in the cave.

### 4.2 Speleothems in Far Hall

In Ignatievskaya cave, the best‐preserved paintings are found in Far Hall. To clarify spatiotemporal relationships between paintings and speleothems, the entire surface of this hall was studied in detail.

The Far Hall is square in plan view (12 × 12 m in size). Its ceiling dips 12–15° toward the east, following the dip of bedding. The floor dips at a somewhat steeper angle due to a massive cone of the surface‐derived debris that extends from a fracture on the western side of the hall. Large slabs of the limestone, detached from the ceiling along the bedding, are abundant on the floor. The eastern and southern walls of the hall are nearly vertical. In contrast, the ceiling and floor nearly merge on the western and northern sides of the hall, meaning that vertical walls are not really present in this area. The height of the hall is 1–5 m.

The surface of the eastern wall of the Far Hall is uneven and exposes a series of bedding planes enlarged by karst dissolution; this surface is not a good “canvas” for painting. In addition, it is mantled by a relatively thick (several cm) flowstone issuing from bedding planes. The upper few‐mm‐thin layers of the flowstone contain abundant soot, giving rise to its black color. Therefore, even if some paintings existed on this wall, they would not be recognizable anymore.

All paintings in the Far Hall are located on its ceiling and southern wall. On the surfaces carrying rock paintings, the flowstone occurs in two varieties: calcite runnels and (sub)mm‐thin layers of translucent calcite forming small patches of several cm^2^ in size. These varieties are closely related and exhibit gradual transitions between each other.

#### 4.2.1 Paintings and speleothems on the ceiling of Far Hall

**Figure 3:**
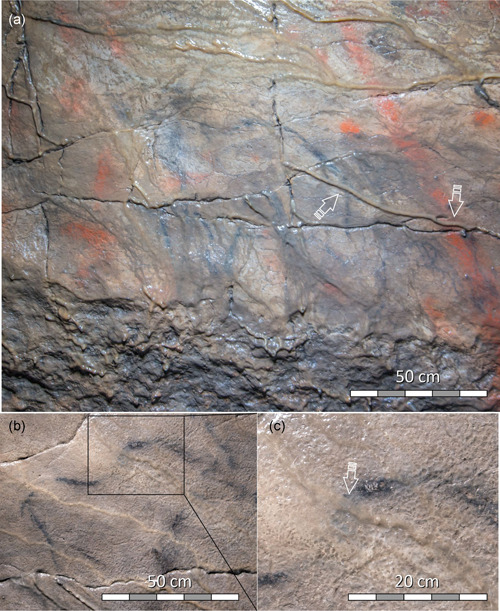
Relationships of flowstone and paintings in Far Hall of Ignatievskaya cave: (a) Black figure *Mammoth* (one of the motifs ^14^C dated by Steelman et al., 2002) and red line (part of the *Bicorn*); (b) Black figure *Horse*; (c) Close‐up of *Horse*'s croup with black paint visible through semitranslucent calcite. Arrows show locations where runnel flowstone was deposited over the pigment [Color figure can be viewed at wileyonlinelibrary.com]

The ceiling is devoid of uniform blackening; instead, it shows abundant vermiculations. It features figures consisting of either red (ochre) or black (charcoal) lines. Many motifs appear to be overgrown by runnel flowstone so that the paint is not visible where flowstone is present (Figure 3a). To assess the possibility that the paintings were made after the flowstone formed, and that the pigment was washed away from protruding surfaces of flowstone runnels, we examined closely the entire surface of the ceiling using optical magnification. We found that flowstone invariably overgrows the paintings—red as well as black (Figure 3). This is obvious from onlapping relationships, where the paint is visible through thinning‐out layers of semitranslucent calcite on the sides of runnels, or where the runnels are generally thin (Figure 3b,c). Nowhere in the Far Hall did we observe a paint, either red or black, on the surface of the flowstone. Occasional black coloration on the surface of the flowstone represents younger soot and never forms part of a painting. Runnel flowstone overgrows the black figure *Mammoth*—one of the motifs that was ^14^C dated by Steelman et al. (2002) (Figure 3a).

#### 4.2.2 Paintings and speleothems on the southern wall of Far Hall

**Figure 4:**
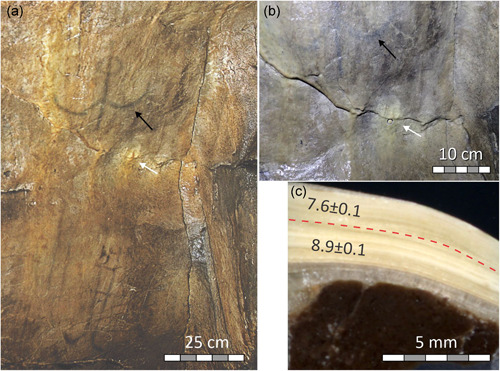
Southern wall of the Far Hall: (a) black (charcoal) *Anchor‐like* sign (black arrow) partly coated by patches of thin flowstone (sampling location shown by white arrow); (b) close‐up of the sampling location (12‐mm‐diameter circular trace of the drill is visible); (c) polished cross‐section of core IGN3 showing bedrock (dark) and laminated flowstone; ^230^Th ages obtained from the two layers (boundary between samples shown by dashed line) given in ka including their uncertainties at the 2*σ* level [Color figure can be viewed at wileyonlinelibrary.com]

The southern wall of the hall features only black (charcoal) motifs (Figure 4a). Flowstone on this steep wall does not form pronounced runnels; instead, it occurs as discontinuous sheets of varying thickness. In some places, the flowstone coating is absent, and in others, it forms mm‐thin translucent layers; in some locations, it thickens and forms 2‐ to 5‐mm‐thin light‐colored patches (Figure 4a,b).

#### 4.2.3 Speleothems on the eastern wall of Far Hall

The eastern wall of Far Hall is the only place in the cave where relatively thick (several cm) flowstone covering several square meters is present. The flowstone is fed by water seeping from bedding partings in the rock; it mantles most of the lower part of the wall.

### 4.3 Speleothems in Large Hall

**Figure 5:**
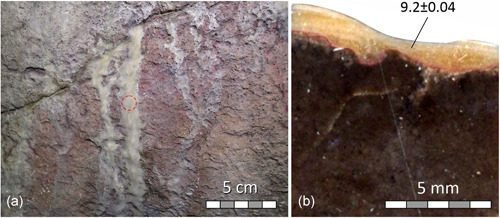
Western wall of the Large Hall: (a) braided runnel flowstone originating in an inclined crack partly coats the red (ochre) painting *New Problematic Mammoth*; position of the ^230^Th sample marked by red circle; (b) polished cross‐section of core IGN6 showing bedrock (dark) and flowstone; note the pigment in between. ^230^Th age is in ka and uncertainty shown at the 2*σ* level [Color figure can be viewed at wileyonlinelibrary.com]

**Figure 6:**
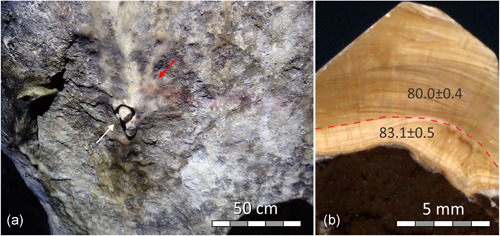
Southern wall of the Large Hall: (a) segment of a runnel flowstone; drill core (not removed) marked by a white arrow; a part of the diffuse red line is visible on the surface of the cave wall and the flowstone (red arrow); (b) polished cross‐section of core IGN7 showing bedrock (dark) and variably laminated flowstone; ^230^Th ages obtained from the two layers (boundary between samples marked by dashed line) are in ka and their uncertainties are shown at the 2*σ* level [Color figure can be viewed at wileyonlinelibrary.com]

Speleothems with identifiable spatiotemporal relationships with paintings were found in two locations in Large Hall. Several flowstone runnels 5−15 mm in width and 70−180 mm in length originate at an inclined fracture on the western wall of the Large Hall (Figure 5a). The flowstone covers the patch of a red pigment known as *New Problematic Mammoth*. Diffuse remnants of a horizontal line made with red paint can be observed on the southern wall of the hall, on the bedrock, and the flowstone surface (Figure 6a). Here, the flowstone runnel was already present when the painting was made.

Petrographic examination of cores taken in this hall revealed that the outer surface of the flowstone was partly destroyed by (most likely) condensation corrosion, which may also be responsible for the destruction of most of the paintings in the hall.

### 4.4 ^230^Th dating of flowstones

Results of the ^230^Th dating of speleothems associated with the paintings are shown in Table 1; detailed results on all dated samples are given in Table S2.

**Table 1:** Abbreviated ^230^Th dating results of samples related to paintings

Spl ID	Site, relation to painting	^238^U (ng/g)	^230^Th/^232^Th	^230^Th/^238^U	δ^234^U^a^	δ^234^U_initial_ b	^230^Th age (a BP) (corrected)
Far Hall							
IGN2‐1	Near *Bicorn*, postpaint	964 ± 3	24 ± 0.5	0.1053 ± 0.0003	187.0 ± 1.9	192.2 ± 2.0	9,715 ± 233
IGN2‐2	Near *Bicorn*, prepaint	616 ± 1	1,213 ± 24	0.6759 ± 0.0016	294.8 ± 1.9	367.1 ± 2.4	77,667 ± 319
IGN3‐1	Near *Anchor*, postpaint, layer 1	735 ± 1	53 ± 1	0.0827 ± 0.0002	206.4 ± 1.5	210.9 ± 1.5	7,547 ± 86
IGN3‐2	Same, layer 2	1,179 ± 3	108 ± 2	0.0944 ± 0.0003	184.1 ± 1.9	188.9 ± 2.0	8,909 ± 59
Large Hall							
IGN6	*New Problematic Mammoth*, postpaint	789 ± 2	376 ± 8	0.1129 ± 0.0004	375.0 ± 1.7	384.9 ± 1.7	9,219 ± 39
IGN7‐1	Diffuse red line, prepaint, layer 1	1,489 ± 4	4,376 ± 89	0.7047 ± 0.0023	320.8 ± 2.0	402.1 ± 2.6	79,983 ± 418
IGN7‐2	Same, layer 2	2,170 ± 7	26,277 ± 575	0.7162 ± 0.0029	307.8 ± 2.1	389.3 ± 2.8	83,137 ± 537

*Note*: Detailed results of ^230^Th dating are presented in Supporting Information, along with the data on speleothems unrelated to paintings. All ratios are activity ratios. Analytical errors are reported at the 95% confidence level. Spl ID, sample identifier. Decay constants: *λ*_238_ = 1.55125 × 10^−10^ (Jaffey et al., 1971), *λ*_234_ = 2.82206 × 10^−6^ and *λ*_230_ = 9.1705 × 10^−6^ (Cheng et al., 2013). BP stands for “Before Present” where the “Present” is defined as the year 1950 C.E.

^a^ δ^234^U = ([^234^U/^238^U]_activity_ – 1) × 1,000.

^b^ δ^234^U_initial_ was calculated based on ^230^Th age (*T*), that is, δ^234^U_initial_ = δ^234^U_measured_ × *e* ^*λ*^_234_^ × T^. Corrected ^230^Th ages assume the initial ^230^Th/^232^Th atomic ratio of 4.4 ± 2.2 × 10^−6^.

#### 4.4.1 Red figure Bicorn (Far Hall, ceiling)

**Figure 7:**
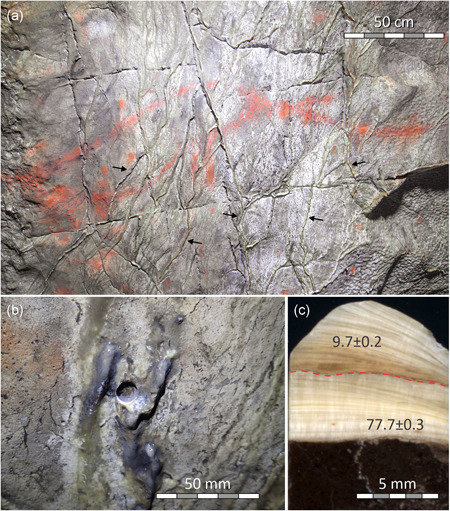
Ceiling of the Far Hall: (a) runnel flowstone (arrows) overgrowing parts of the ochre painting *Bicorn*; (b) segment of a runnel flowstone cored for ^230^Th dating (dark gray coloration on the surface of flowstone is caused by soot resulting from visits of the hall in 19th to 20th century); (c) polished cross‐section of core IGN2 showing bedrock (dark) and laminated flowstone; ^230^Th ages obtained from the two distinct layers (petrographically defined hiatus is marked by dashed line) given in ka including their uncertainties at the 2*σ* level [Color figure can be viewed at wileyonlinelibrary.com]

Runnel flowstone partly overgrows the red figure *Bicorn* on the slightly inclined ceiling of the Far Hall (Figure 7a). The flowstone depositional setting is rather simple, and the runnel could be traced from places where it overlays the paintings to those where the wall surface is not painted. On the sides of the runnel, where calcite layers thin‐out, the pigment can be seen through semitranslucent calcite coating. This ascertains that pigment had not been removed from the speleothem surface but was overgrown by flowstone.

Two mini‐cores, IGN1 and IGN2 (Figure 7b), were drilled through the flowstone less than 50 cm from paintings overgrown by it. Petrographic examination of the cores revealed two stages of flowstone deposition, separated by a clearly identifiable hiatus (Figure 7c). In one core (IGN2), the two layers were thick enough to be ^230^Th‐dated separately. They returned ages of 77.7 ka and 9.7 ka BP. As there is no reason to consider such an old age for artistic activity in the cave, the most likely interpretation is that the paintings were made on the ceiling where 2‐ to 4‐mm‐thin 77.7 ka‐old flowstone was present, which were then overgrown by a second generation of flowstone in the early Holocene.

#### 4.4.2 Black anchor‐like sign (Far Hall, southern wall)

One of the black charcoal motifs on the southern wall of the Far Hall has a conspicuous anchor‐like shape (Figure 4a). Core IGN3 was drilled through 4 to 5‐mm‐thin patch of flowstone c. 15 cm from the motif (Figure 4b). The flowstone is undoubtedly younger than the motif because translucent thinning‐out layers of the flowstone coat the left part of the figure. Petrographic examination revealed two growth layers of distinct colors (Figure 4c), but there is no evidence of a hiatus between the layers. Being too thin to be dated separately, the older layer was sampled together with the younger yellowish one; another date was obtained on the outer part of the younger layer. As the mixture produced an age that is only slightly older than the outer part of the flowstone (c. 8.9 ka and 7.6 ka BP, respectively), we conclude that the earliest translucent layer also has an early Holocene age.

#### 4.4.3 Red figure new problematic mammoth (Large Hall, western wall)

Petrographic examination of core IGN6 drilled through one of the runnels overgrowing the motif New Problematic Mammoth showed that the pigment was applied on a barren cave wall and was later overgrown by flowstone (Figure 5b). The flowstone returned an early Holocene age of c. 9.2 ka BP.

#### 4.4.4 Red line (Large Hall, Southern wall)

Two subsamples from the core drilled through an older flowstone, on which the red paint was applied, returned stratigraphically consistent ages of c. 83.1 and c. 80.0 ka BP (Figure 6b).

#### 4.4.5 Speleothems unrelated to paintings

##### 4.4.5.1 Stalagmite (Far Hall)

**Figure 8:**
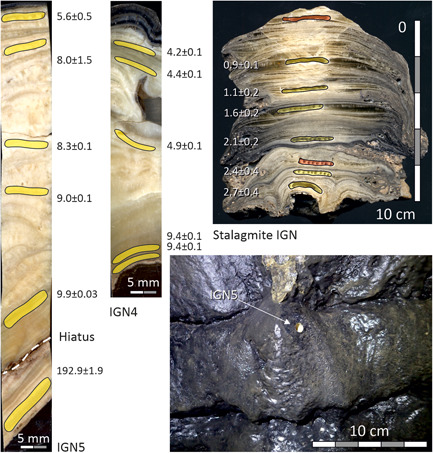
Polished sections of cores IGN4, IGN5, and stalagmite IGN showing locations of subsamples for ^230^Th dating (yellow—successful; red—failed). ^230^Th ages are given in ka including their uncertainties at the 2*σ* level. Sampling site of core ING5 is shown in the lower right photo [Color figure can be viewed at wileyonlinelibrary.com]

Core IGN4 was drilled along the axis of a c. 50‐mm‐tall stalagmite, formed on a boulder in the eastern part of the Far Hall. The stalagmite formed where water that had flowed along the ceiling and formed a runnel flowstone dripped on the floor. This stalagmite, therefore, is an equivalent of runnel flowstone IGN2 sampled at the chamber's ceiling (Figures S1–2). Five samples from this stalagmite yielded stratigraphically consistent Holocene ages of c. 9.4–4.2 ka BP (Figure 8).

##### 4.4.5.2 Flowstone (eastern wall of Far Hall)

Core IGN5 was drilled through thick flowstone deposited on the eastern wall of the Far Hall. A c. 1‐cm‐thick layer of old flowstone (193 ka BP) is present at the base, which is separated from a c. 7‐cm‐thick overgrowth by a clearly identifiable hiatus. Five samples taken above the hiatus returned stratigraphically consistent ages ranging from 9.9 to 5.6 ka BP (Figure 8).

##### 4.4.5.3 Stalagmite (Large Hall)

Stalagmite IGN was provided to us by biologist N. Erokhin, who collected it (not in situ) in the southern part of Large Hall. Six samples from this stalagmite yielded stratigraphically consistent late Holocene ages of 2.7–0.9 ka BP (Figure 8).

## 5 DISCUSSION

### 5.1 Evaluation of the ^230^Th dating results

Holocene flowstones from Ignatievskaya cave contain 130 to 2000 ng/g ^238^U and 0.5 to 12.8 ng/g ^232^Th. These values allow for relatively high‐precision dating with minimal shifts in detritally corrected ages and relative age uncertainties in the order of c. 1%. In contrast to flowstone, the late Holocene stalagmite IGN has lower ^238^U concentrations (95–175 ng/g) while maintaining similar levels of ^232^Th (1.1–5.5 ng/g), resulting in a mean relative age uncertainty of c. 15%.

Where multiple dates were obtained on a given sample, the ages are in a stratigraphic order, which supports the notion of closed‐system behavior of U and Th in these speleothems.

When applying ^230^Th dating to speleothems overgrowing paintings, we are interested in the time when the flowstone began to form (*terminus ante quem*). One limitation of our dating approach in Ignatievskaya cave is that the postpaint flowstone is thin (2−3 mm). In two cases, the entire thickness of the flowstone layers present in the core had to be milled to obtain enough material for dating (Figures 5b and 7c). Such bulk ages represent a *minimum* estimate of the flowstone age.

We were able to constrain the time of initiation of calcite deposition for sample IGN3, for which two layers were dated (Figure 4c). A growth model based on these data indicates that flowstone growth commenced at c. 9.4 ka and ended at c. 6.9 ka (Figures S3–1 and S3–2).

The Holocene age of speleothem deposition in the Far Hall of Ignatievskaya cave is further supported by growth models for samples IGN4 and IGN5 (Figures S3–3 and S3–5), which indicate that growth started around 10.1 ka and terminated at c. 4.0 ka (Figures S3–4 and 3–6). Although these dates are unrelated to paintings, they are relevant because the samples represent lateral equivalents of the postpaint runnel flowstone (Figures S1–2).

### 5.2 The ^230^Th time window

The youngest prepaint flowstone was dated in two cores and yielded c. 77.7 ka BP (IGN2) and c. 80.0 ka BP (IGN7). Being single dates, these ages are maximum age estimates. The latest prepaint episode of flowstone growth, thus, relates to Marine Isotope Stage (MIS) 5a. Most of the ceiling in the Far Hall is barren; therefore, the amount of calcite deposited at this time was extremely small. A still older growth episode, corresponding to MIS 7a (c. 193 ka BP), was documented locally at the base of flowstone IGN5.

The postpaint flowstone in both the Far Hall and the Large Hall is of Holocene age. The oldest (closest to the age of paintings) dates are 9.7 ka BP (IGN2) and 9.2 ka BP (IGN6). These two dates were obtained from individual layers and are, therefore, minimum age estimates. The date of 9.4 ka BP corresponds to the onset of flowstone growth in sample IGN3 (derived from the growth model; Figures S3–2). A small stalagmite and relatively thick flowstone sheet formed in the Far Hall between c. 10.1 ka BP and c. 4.0 ka BP (IGN4 and IGN5); stalagmite IGN from Large Hall grew from c. 2.7 ka BP until the present (Figures S3–8).

Our dating results indicate that flowstone did not form in Ignatievskaya cave for an extended period of time between c. 77.7 ka BP (MIS 5a) and c. 10.1 ka BP. Paintings on the walls of Ignatievskaya cave were made during this time window. The latter is much larger than that determined for paintings in Shulgan‐Tash cave, located some 200 km to the south. There, short episodes of prepaint flowstone growth occurred in the late part of MIS 3 around 30 ka BP, and calcite growth resumed (covering the paintings) earlier, during the Bølling–Allerød interstadial at c. 14.5 ka BP (Dublyansky et al., 2018).

### 5.3 Comparison of ^230^Th and ^14^C dates from the cultural layer

**Figure 9:**
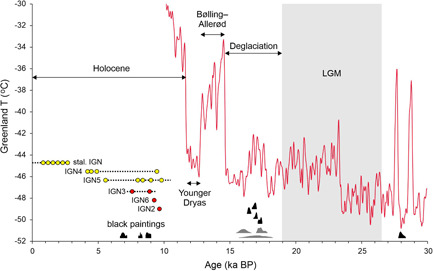
Comparison of ^14^C and ^230^Th dates from Ignatievskaya cave in context of paleoclimate. ^14^C dates are shown as 95.4% probability distributions. Dates from the cultural layer: gray—conventional; black—AMS (Dublyansky & Shirokov, 2020; Dublyansky et al., 2021); AMS dates from black paintings: Black (Steelman et al., 2002). ^230^Th dates: Red circles—flowstone postdating paintings (minimum ages); yellow circles—not associated with paintings. The dotted line indicates continuity of speleothem growth; it starts from the most likely start of growth time, determined by age modeling. Dates are shown in the context of the temperature in Greenland obtained from NGRIP core (Kindler et al., 2014). Placement of ages on the figure is unrelated to temperatures. AMS, accelerator mass spectrometry; BP, before present; LGM, last glacial maximum; NGRIP, North Greenland Ice Core Project [Color figure can be viewed at wileyonlinelibrary.com]

Several radiocarbon dates were reported from the cultural layer found in archaeological pits II, III, and IV excavated in the cave (Figure 2; Table S4). These ^14^C dates are shown in Figure 9 along with the results of the ^230^Th dating of the postpaint flowstone. We note here that the cultural layer in Ignatievskaya cave is related to the paintings due to clumps of ochre found in this layer (Petrin, 1992, p. 96). Assuming that these ^14^C dates correspond to the time of artistic activity in the cave, the ^230^Th dates are consistent with (i.e., younger than) the ^14^C dates.

Dating of the cultural layer in Ignatievskaya cave is challenging. Conditions in the aphotic zone of the cave (the Large Hall and the Low Passage), where archaeological excavations uncovered the Paleolithic cultural layer (pits I through IV), have remained rather dry since the Upper Pleistocene; there is no evidence of even episodic ponds or streams. Accumulation of sediment occurred subaerially (Stefanovsky, 2002) at very low sedimentation rates, giving rise to only weakly consolidated deposits. The cultural layer in this cave is thin, located very close to the cave floor (0.05–0.25 m), and is typically covered by loose, powdery sediment, representing a mix of the Holocene and the remobilized Upper Pleistocene material.

Furthermore, the sediment in Ignatievskaya cave was subject to some mixing. Archaeologists working in the cave in the 1980s reported that many Paleolithic artifacts (stone tools, decorations, bones) were found directly on the cave floor (Petrin, 1992). Also, bones of spotted hyena (*Crocuta c. spelaea*), a species that extirpated in Northern Eurasia at around c. 40 ka (Stuart & Lister, 2014), were found on the cave floor. These bones, which yielded (uncalibrated) ^14^C ages of c. 40.2, c. 44.3, >40.1, and >62.3 ka BP (Stuart & Lister, 2014), were most likely derived from deeper sediment layers, where they are abundant (Smirnov et al., 1990). Sediment mixing may have started already in the Paleolithic. For example, two bone samples from a well‐defined cultural layer in Pit II (depth: 0.06–0.08 m and 0.12–0.14 m) returned ^14^C ages ranging from c. 25.6 to c. 24.4 cal ka BP (Dublyansky et al., 2021; Table S4), which are much older than the ^14^C dates obtained from charcoal samples from the same layer (c. 13.2–11.6 and c. 17.7–15.7 cal ka BP; Table S4). Apparently, the Upper Pleistocene bones from sediments underneath the cultural layer were incorporated into the cultural layer at the time of its formation.

### 5.4 Comparison of ^230^Th dates and ^14^C dates from black paintings

In contrast to the ^14^C data of the cultural layer, direct ^14^C dates of black paintings (Steelman et al., 2002; Table S5) in Far Hall are not consistent with the new ^230^Th dates (Figure 9). Black paintings (c. 9.0−6.5 cal ka BP) appear younger than the flowstone that overgrew both the black (min. 9.6 ka BP) and red paintings (min. 9.7 ka BP) in the same hall.

We note that none of our ^230^Th dates refer exactly to the same motif from which the ^14^C dates of Steelman et al. (2002) were obtained. In fact, only one of our samples, IGN3, constrains the antiquity of the black painting; others, IGN2 and IGN6, provide *terminus ante quem* for the red (ochre) paintings. We note, however, that the red motif *Bicorn* (our sample IGN2) is located very close to the black motif *Mammoth* dated by Steelman et al. (2002), and that both figures, red and black, are overgrown by flowstone runnels identical to the one sampled as IGN2 (Figure 3a).

In view of the new ^230^Th dates, the ^14^C ages of black paintings appear to be erroneous (too young). At the time of publication, Steelman et al. (2002) acknowledged that their dates appear “unexpectedly recent” (p. 347). They carefully evaluated the data and discarded many possible reasons for obtaining ^14^C ages younger than the “true” age of black paintings. Furthermore, they offered four explanations of the inconsistency, three of which questioned the presumed Paleolithic antiquity of paintings and one suggested a mixing of two painting episodes: “There was repainting of an older image, such that younger overpainting and original painting charcoal combines to give a meaningless radiocarbon age, more recent that the initial painting event” (p. 348). As it is known, in the 19th century, the Far Hall was used for years as a hermitage by Orthodox monk Ignatiy, and after his death, it was frequented by his followers. Visits to the hall involving candles and torches continued through the 20th and into the early 21th century. Given that paintings are readily visible and accessible on the ceiling of the hall, the explanation offered by Steelman and coauthors is not unreasonable.

### 5.5 Artistic activity in Ignatievskaya cave: Paleoclimatic context

Available geochronological information from Ignatievskaya cave is summarized in Figure 9 in the context of northern hemisphere paleoclimate. The ^14^C dates from the cultural layer indicate that the cave was visited by an ancient man during deglaciation that followed the last glacial maximum (LGM). A single ^14^C date suggests that the cave could have been visited also before the LGM (this result requires validation). Multiple lines of evidence converge on the conclusion that the cave was visited and used for artistic activity in Upper Paleolithic times.

#### 5.5.1 Iconography

Paintings of the representative of the Pleistocene fauna, the mammoth, are found in the parietal art in the cave, along with paintings of horses. Other ice‐age animals are also present; however, their rendering is commonly not realistic; rather, they feature as parts of the composite, sometimes unreal motifs, such as rhinoceros‐ and mammoth‐like figures, a figure with the body of a camel and a horse with horns. All animals are known to have inhabited Southern Ural during the Upper Pleistocene, and Upper Paleolithic paintings of mammoths, woolly rhinoceros, horses, and a camel are known from the Shulgan‐Tash cave, some 200 km to the south. The animals pictured in Ignatievskaya cave are distinct from post‐Paleolithic open‐air rock art in the area, featuring elks, deer, roe, waterbirds, and occasional bears. The very repertoire of the paintings in Ignatievskaya cave, thus, points toward their Paleolithic age, as the large mammals of the “mammoth fauna” were driven to extinction in Southern Ural at the end of the Pleistocene (Danukalova et al., 2020; Kosintsev and Bachura, 2013).

#### 5.5.2 Paleozoology

In addition to charcoal and archaeological artifacts, abundant bone remnants were reported from the cultural layer. Identified species include mountain hare (*Lepus timidus*), steppe marmot (*Marmota bobak*), Arctic fox (*Vulpes lagopus*), red fox (*Vulpes vulpes*), horse (*Equus ferus*), weasel (*Mustela nivalis*), ermine (*Mustela erminea*), steppe polecat (*Putorius* sp.), wolf (*Canis lupus*), bison (*Bison priscus*), and saiga antelope *(Saiga tatarica)*. Small mammals are represented by narrow‐headed vole (*Lasiopodomys gregalis*), root vole (*Microtus oeconomus*), field vole (*Microtus agrestis*), steppe pika (*Ochotona* sp.), palearctic collared lemming (*Dicrostonyx gulielmi*), russet ground squirrel (*Citellus major*), gray hamster (*Cricetulus migratorius*), Eurasian water vole (*Arvicola terrestris*), and Eversmann's hamster (*Allocricetus eversmanni*) (Smirnov et al., 1990). The osteological material, thus, features species adapted to a harsh climate and tundra to forest–tundra landscapes.

It is to be noted that abundant bones and coprolites of cave bear (*Spelaearctos spelaeus*) form a pronounced horizon in the cave sediments just c. 0.25 m below the cultural layer and in deeper strata. Although the age of these bones is poorly constrained (the only sample of cave bear bone removed from a depth of 0.9−1.0 m in Pit II returned an indefinite ^14^C age of over c. 27.6 ka; IEMEA–25; Smirnov et al., 1990), it is presently accepted that the cave bear became extinct in the Ural Mountains around 41 ka BP (Pacher & Stuart, 2009).

#### 5.5.3 Palynology

Pollen in the cultural layer are poorly preserved. Of those present, 97% represent herbaceous plants, of which the most abundant group is *Asteraceae* (including *Artemisia*). Sedges (*Carex* sp.) and grasses (*Gramineae*) are rather abundant. Rare arboreal pollen grains are represented by shrub birch (*Betula*) and willow (*Salix*) (Panova & Bykova, 1992). Twenty‐six charcoal fragments were examined to determine the type of wood that was used by Paleolithic man to make torches (Shiyatov, 1992). Most of the fragments were from the pine (*Pinus*) and only three were from alder (*Alnus*). The paleobotanical evidence indicates a sparse vegetation of the periglacial type, characteristic of a dry and strongly continental climate. The landscape most likely featured cold‐steppe and forest‐steppe, with small patches of forests.

#### 5.5.4 Permafrost

The period of Paleolithic cave visits, indicated by ^14^C dates of the cultural layer, broadly coincides with the “Last Permafrost Maximum” (c. 25–17 ka; Vandenberghe et al., 2014). At that time, the southern boundary of equilibrium permafrost in this part of Eurasia was at about 48°N. Ignatievskaya cave (53.5°N), thus, resided in the area of permanently frozen ground.

It has been shown that no stalagmite deposition occurred between c. 73 and 7.7 ka (i.e., during most of MIS 4 and the entire MIS 3 and 2) in two other Southern Ural caves, Shulgan‐Tash and Victoria (Dublyansky et al., 2018). This is consistent with the interruption of speleothem growth in Ignatievskaya cave between c. 78 and 10 ka. Permafrost conditions are known to have interrupted speleothem growth in Siberian caves (Vaks et al., 2013). Similarly, the gap in speleothem growth in Ignatievskaya cave is attributed to permafrost in Southern Ural. As Ignatievskaya cave is located c. 200 km to the north of Shulgan‐Tash and Victoria caves, at a similar elevation, it is reasonable to assume that at this time, the cave was also located in the zone of continuous permafrost, where the thickness of perennially frozen ground reached c. 200 m and the mean annual air temperature was between −5°C and −3°C (Velichko et al., 2002; Vandenberghe et al., 2014). The conclusion reached for Shulgan‐Tash cave that “… during the time of artistic activity … cave was an inhospitable place with below‐zero air temperatures throughout the year and no dripping or running water. The paintings were made by Paleolithic artists on cave walls that were dry; our data indicate that the dryness was due to the freezing temperatures” (Dublyansky et al., 2018, p. 5) is also valid for Ignatievskaya cave.

## 6 CONCLUSIONS

^230^Th dates obtained on flowstone that formed before and after the red and black paintings in Ignatievskaya cave constrain the age of the artistic activity between c. 78 and c. 10 ka. This long period of time during which speleothems did not form reflects cold climatic conditions and the development of permafrost in Southern Ural. This interpretation is further supported by the available paleozoological and palynological data from the cultural layer in the cave, featuring species adapted to harsh cold climates.

The ^14^C dates obtained from bones and charcoal of the cultural layer in the deep, aphotic zone of the cave (Dublyansky et al., 2021; Petrin, 1992; Smirnov et al., 1990) are broadly consistent with the ^230^Th dating results. They place the cave visits (and, by extension, the creation of cave art) at c. 18.3−15.8 cal ka BP, that is, the late glacial period.

Three ^14^C dates obtained directly from charcoal paintings in the Far Hall of the cave (Steelman et al., 2002) appear too young and are inconsistent with the ^230^Th dates. These ^14^C dates are younger than the flowstone that formed after both red and black paintings in the hall were made.

## CONFLICT OF INTERESTS

The authors declare that there are no conflict of interests.

### PEER REVIEW

The peer review history for this article is available at https://publons.com/publon/10.1002/gea.21851.

## Supporting information

Supporting information.

## Data Availability

The authors confirm that the data supporting the findings of this study are available in the article Supporting Information Material.
