# Supplementary material for: 230Th dating of flowstone from Ignatievskaya Cave, Russia: Age constraints of rock art and paleoclimate inferences
Source: Geoarchaeology. 2021 Feb 24;36(3):532–45. doi: 10.1002/gea.21851 (PMC8048586; doi:10.1002/gea.21851)
Supplement: Supplementary file 1 — Supporting information. [file GEA-36-532-s001.docx]

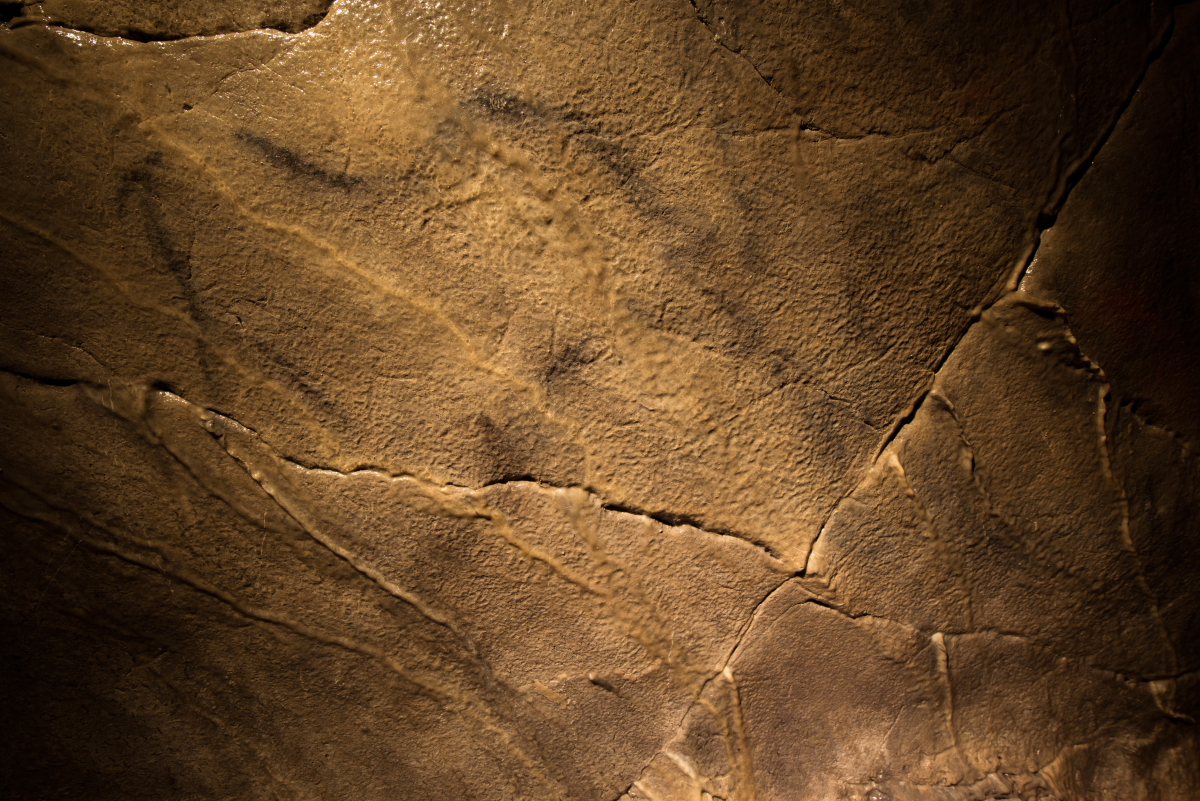


Supporting Information for:

^230^Th dating of flowstone from Ignatievskaya Cave, Russia: Age constraints of rock art and paleoclimate inferences

By: Dublyansky Y., Shirokov V., Moseley G.E., Kosintsev P.A., Edwards R.L., Spötl C.

*Cover photo*: Ignatievskaya cave (Ural, Russia), black figure *Horse* (figure length c. 0.60 m).
© Robbie Shone

# Section S1. Explanatory drawings


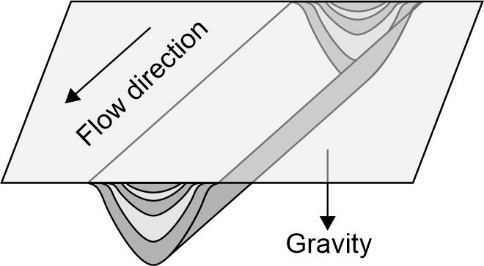


Fig. S1-1. Schematic rendering of the runnel flowstone forming on the ceiling of the cave. Depending on the slope of the ceiling and the water flow regime, these inverted-crest accretions may become more or less flat. At shallow angles, very thin calcite may also coat the ceiling adjacent to the runnel.


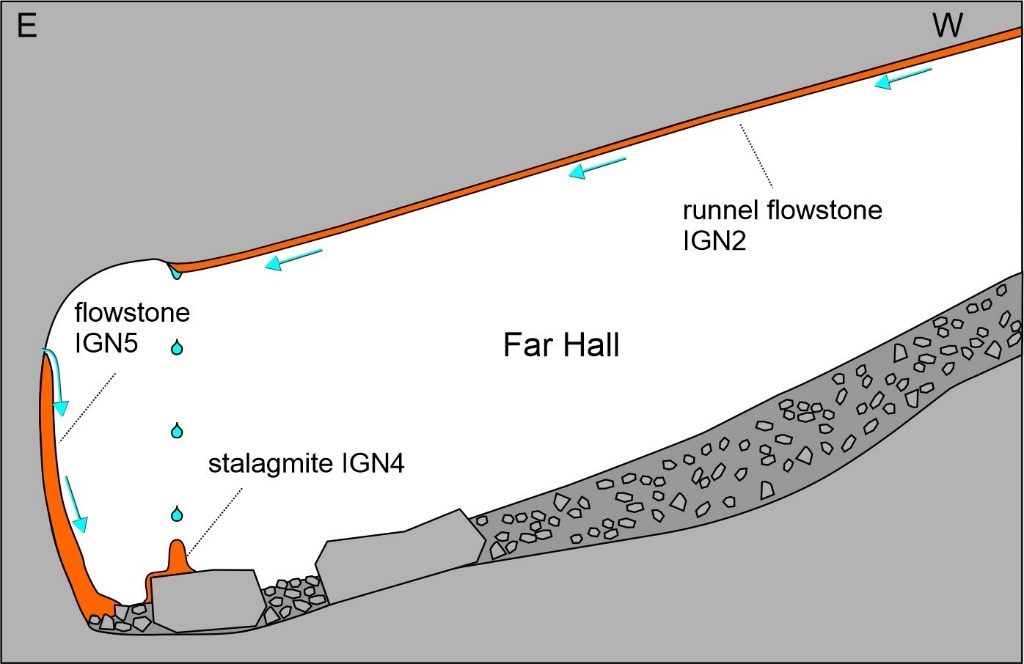


Fig. S1-2. Schematic drawing showing relationships between speleothems sampled in the Far Hall of Ignatievskaya cave. Water films and drops are shown in blue. Stalagmite IGN4 is a homologue to runnel flowstone IGN2 (both formed from water flowing along the roof of the hall). Flowstone IGN5 formed from water discharging from a separate fracture in the eastern wall of the hall.

# Section S2. ^230^Th results of samples from Ignatievskaya cave

Table S2

| **Sample** | **DFB** | **^238^U** | | **^232^Th** | | **^230^Th / ^232^Th** | | **δ^234^U*** | | **^230^Th / ^238^U** | | **^230^Th Age (yr)** | | **δ^234^U_Initial_**** | | **^230^Th Age (yr BP)** | |
| --- | --- | --- | --- | --- | --- | --- | --- | --- | --- | --- | --- | --- | --- | --- | --- | --- | --- |
| **ID** | **(mm)** | **(ng/g)** | | **(ng/g)** | | **(atomic x10^-6^)** | | **(measured)** | | **(activity)** | | **(uncorrected)** | | **(corrected)** | | **(corrected)** | |
| IGN1^+^ | 2 | 566.4 | ± 1.0 | 15.4 | ± 0.3 | 175 | ± 4 | 257.0 | ± 1.6 | 0.2878 | ±0.0007 | 28,099 | ± 84 | 277.8 | ± 1.8 | **27,412** | **±446** |
| IGN2-1 | 2 | 964.3 | ± 2.5 | 12.8 | ± 0.3 | 131 | ± 3 | 187.0 | ± 1.9 | 0.1053 | ±0.0003 | 10,108 | ± 36 | 192.2 | ± 2.0 | **9,715** | **±233** |
| IGN2-2 | 6 | 616.3 | ± 1.3 | 1.1 | ± 0.0 | 6,507 | ± 131 | 294.8 | ± 1.9 | 0.6759 | ±0.0016 | 77,772 | ± 318 | 367.1 | ± 2.4 | **77,667** | **±319** |
| IGN3-1 | 1 | 735.1 | ± 1.3 | 3.5 | ± 0.1 | 283 | ± 6 | 206.4 | ± 1.5 | 0.0827 | ±0.0002 | 7,731 | ± 25 | 210.9 | ± 1.5 | **7,547** | **±86** |
| IGN3-2 | 3 | 1,179.4 | ± 3.1 | 3.2 | ± 0.1 | 579 | ± 12 | 184.1 | ± 1.9 | 0.0944 | ±0.0003 | 9,043 | ± 36 | 188.9 | ± 2.0 | **8,909** | **±59** |
| IGN4-1 | 3 | 2,048.4 | ± 7.7 | 3.7 | ± 0.1 | 894 | ± 18 | 176.0 | ± 2.2 | 0.0984 | ±0.0004 | 9,510 | ± 46 | 180.7 | ± 2.3 | **9,397** | **±55** |
| IGN4-2 | 1 | 2,018.6 | ± 13.5 | 2.2 | ± 0.0 | 1,489 | ± 35 | 180.6 | ± 3.9 | 0.0989 | ±0.0012 | 9,521 | ± 125 | 185.5 | ± 4.0 | **9,426** | **±127** |
| IGN4-3 | 41 | 365.0 | ± 0.3 | 2.2 | ± 0.0 | 139 | ± 3. | 246.1 | ± 1.7 | 0.0498 | ±0.0005 | 4,439 | ± 43 | 249.1 | ± 1.8 | **4,232** | **±106** |
| IGN4-4 | 38 | 373.6 | ± 0.3 | 0.5 | ± 0.0 | 615 | ± 14 | 246.2 | ± 1.5 | 0.0503 | ±0.0005 | 4,489 | ± 42 | 249.3 | ± 1.5 | **4,388** | **±48** |
| IGN4-5 | 22 | 311.2 | ± 0.2 | 0.5 | ± 0.0 | 530 | ± 12 | 249.9 | ± 1.7 | 0.0564 | ±0.0006 | 5,029 | ± 52 | 253.5 | ± 1.8 | **4,919** | **±60** |
| IGN5-1 | 3 | 198.3 | ± 0.2 | 18.0 | ± 0.4 | 186 | ± 4 | 189.8 | ± 1.4 | 1.0274 | ±0.0021 | 194,972 | ± 1253 | 327.2 | ± 3.0 | **192,892** | **±1,881** |
| IGN5-2 | 65 | 193.6 | ± 0.3 | 6.1 | ± 0.1 | 39 | ± 1 | 303.0 | ± 2.3 | 0.0744 | ±0.0007 | 6,393 | ± 59 | 307.9 | ± 2.4 | **5,620** | **±502** |
| IGN5-3 | 59 | 126.9 | ± 0.2 | 11.7 | ± 0.2 | 21 | ± 0 | 293.4 | ± 2.4 | 0.1152 | ±0.0010 | 10,137 | ± 93 | 300.1 | ± 2.7 | **7,988** | **±1476** |
| IGN5-4 | 40 | 318.9 | ± 0.4 | 1.9 | ± 0.0 | 245 | ± 5 | 197.9 | ± 1.8 | 0.0905 | ±0.0005 | 8,550 | ± 52 | 202.7 | ± 1.8 | **8,334** | **±116** |
| IGN5-5 | 31 | 663.0 | ± 1.1 | 4.9 | ± 0.1 | 218 | ± 4 | 186.7 | ± 1.7 | 0.0970 | ±0.0003 | 9,279 | ± 34 | 191.5 | ± 1.8 | **9,031** | **±131** |
| IGN5-6 | 9 | 1,857.6 | ± 4.4 | 0.5 | ± 0.0 | 6,434 | ± 131 | 174.7 | ± 1.6 | 0.1024 | ±0.0003 | 9,921 | ± 31 | 179.7 | ± 1.7 | **9,846** | **±32** |
| IGN6 | 1 | 789.1 | ± 1.5 | 0.7 | ± 0.0 | 2,017 | ± 42 | 375.0 | ± 1.7 | 0.1129 | ±0.0004 | 9,306 | ± 36 | 384.9 | ± 1.7 | **9,219** | **±39** |
| IGN7-1 | 1 | 1,489.1 | ± 4.4 | 0.7 | ± 0.0 | 23,479 | ± 476 | 320.8 | ± 2.0 | 0.7047 | ±0.0023 | 80,061 | ± 418 | 402.1 | ± 2.6 | **79,983** | **±418** |
| IGN7-2 | 5 | 2,170.1 | ± 7.4 | 0.2 | ± 0.0 | 140,984 | ± 3,085 | 307.8 | ± 2.1 | 0.7162 | ±0.0029 | 83,207 | ± 537 | 389.3 | ± 2.8 | **83,137** | **±537** |
| IGN01-2 | 56 | 137.1 | ± 0.2 | 3.1 | ± 0.1 | 19 | ± 1 | 751.4 | ± 2.4 | 0.0255 | ±0.0006 | 1,598 | ± 37 | 754.0 | ± 2.5 | **1,152** | **±270** |
| IGN01-16 | 42 | 135.9 | ± 0.1 | 1.1 | ± 0.0 | 33 | ± 1 | 747.2 | ± 1.7 | 0.0169 | ±0.0004 | 1,061 | ± 22 | 749.1 | ± 1.8 | **853** | **±101** |
| IGN01-24 | 34 | 106.1 | ± 0.1 | 1.5 | ± 0.0 | 27 | ± 1 | 766.1 | ± 1.7 | 0.0228 | ±0.0006 | 1,418 | ± 37 | 768.7 | ± 1.8 | **1,121** | **±166** |
| IGN01-30 | 28 | 145.0 | ± 0.1 | 2.6 | ± 0.1 | 29 | ± 1 | 765.4 | ± 2.0 | 0.0311 | ±0.0005 | 1,935 | ± 34 | 769.0 | ± 2.0 | **1,575** | **±209** |
| IGN01-39 | 19 | 93.7 | ± 0.1 | 1.9 | ± 0.0 | 33 | ± 1 | 818.5 | ± 2.6 | 0.0405 | ±0.0010 | 2,452 | ± 60 | 823.4 | ± 2.6 | **2,055** | **±240** |
| IGN01-49 | 9 | 174.4 | ± 0.2 | 5.5 | ± 0.1 | 26 | ± 1 | 829.2 | ± 2.7 | 0.0490 | ±0.0009 | 2,957 | ± 52 | 834.9 | ± 2.8 | **2,389** | **±358** |
| IGN01-53 | 3 | 119.8 | ± 0.1 | 4.1 | ± 0.1 | 27 | ± 1 | 909.7 | ± 2.0 | 0.0563 | ±0.0007 | 3,255 | ± 38 | 916.7 | ± 2.2 | **2,670** | **±368** |

Decay constants: λ_238_ = 1.55125×10^-10^ (Jaffey et al., 1971), λ_234_ = 2.82206×10^-6^ (Cheng et al., 2013), λ_230_ = 9.1705×10^-6^ (Cheng et al., 2013). *δ^234^U = ([^234^U/^238^U]_activity_ – 1) ×1000; ** δ^234^U_initial_ was calculated based on ^230^Th age (T), i.e., δ^234^U_initial_ = δ^234^U_measured_ × e^λ234×T^. Corrected ^230^Th ages assume the initial ^230^Th/^232^Th atomic ratio of 4.4 ±2.2 x10^-6^. DFB – distance from base; ^+^ – mixed age; BP stands for “Before Present” where the “Present” is defined as the year 1950 C.E.

# Section S3. Growth models for samples IGN3, IGN4, IGN5, and stalagmite IGN

The growth models presented in this section were constructed using OxCal 4.4 (Bronk Ramsey, 2008; Bronk Ramsey & Lee, 2013).

## Flowstone IGN3


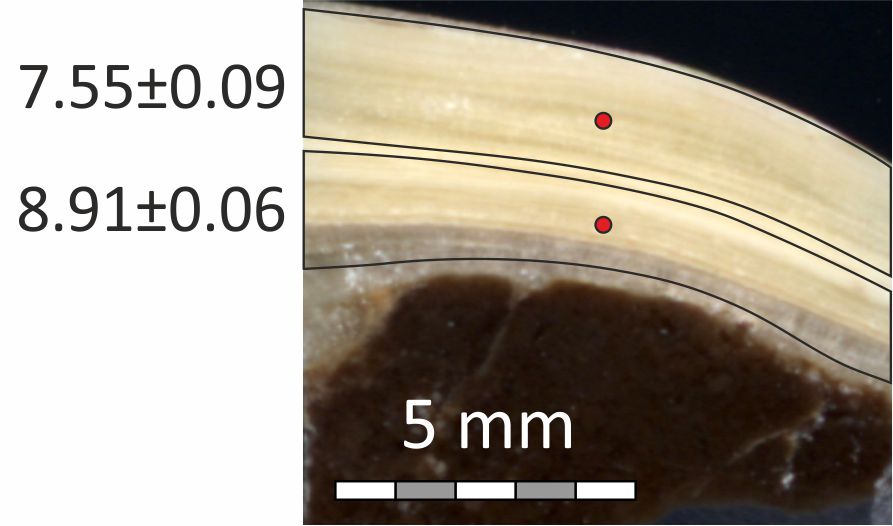
Fig. S3-1. Sample IGN3. Outlines of layers taken for dating are shown. Red dots indicate positions used to define distances from base of the crust. Numbers are ^230^Th ages ±2σ uncertainty (ka BP).


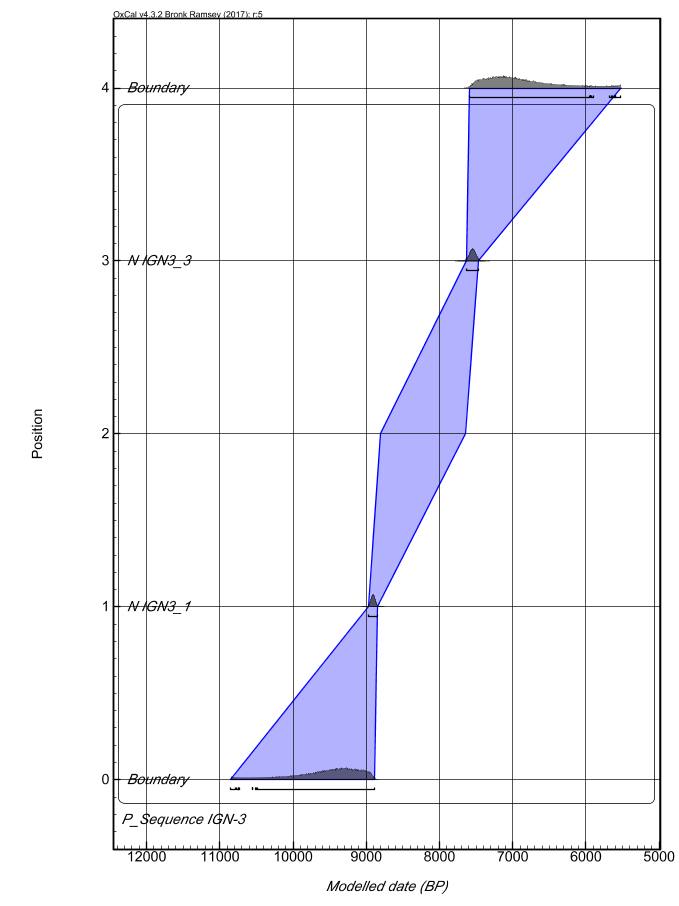


Fig. S3-2. Growth model for sample IGN3. IGN3_1 and IGN3_3 – measured ages. Grey areas depict 0.945 probability distribution at fixed positions. Blue area depicts 0.945 probability boundaries.

## Stalagmite IGN4

Stalagmite IGN4 is a homologue to runnel flowstone IGN2 (both formed from water flowing along the roof of the hall; see Fig. S1-2).


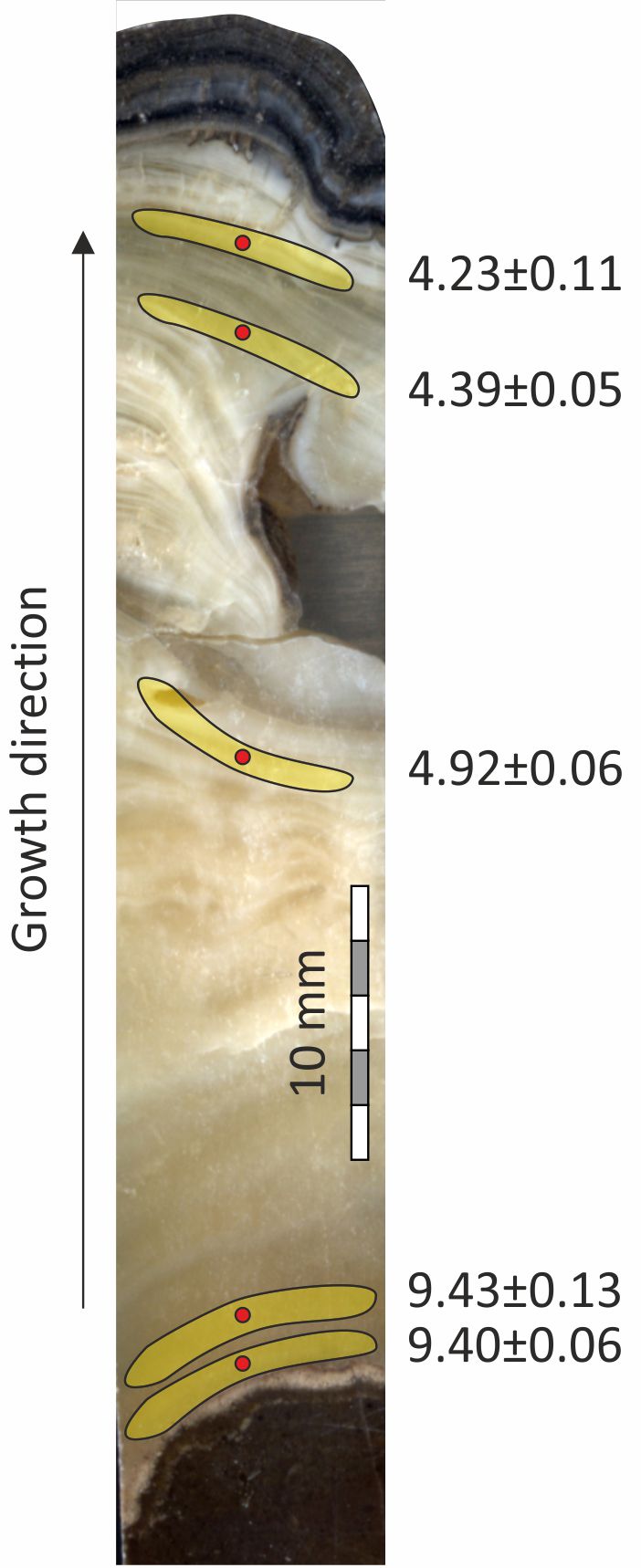


Fig. S3-3. Sample IGN4. Outlines of layers taken for dating are shown. Red dots indicate positions used to define distances from base of the stalagmite. Note that the upper 2−3 mm-thin layer is stained black (likely due to soot particles). Numbers are ^230^Th ages ±2σ uncertainty (ka BP).


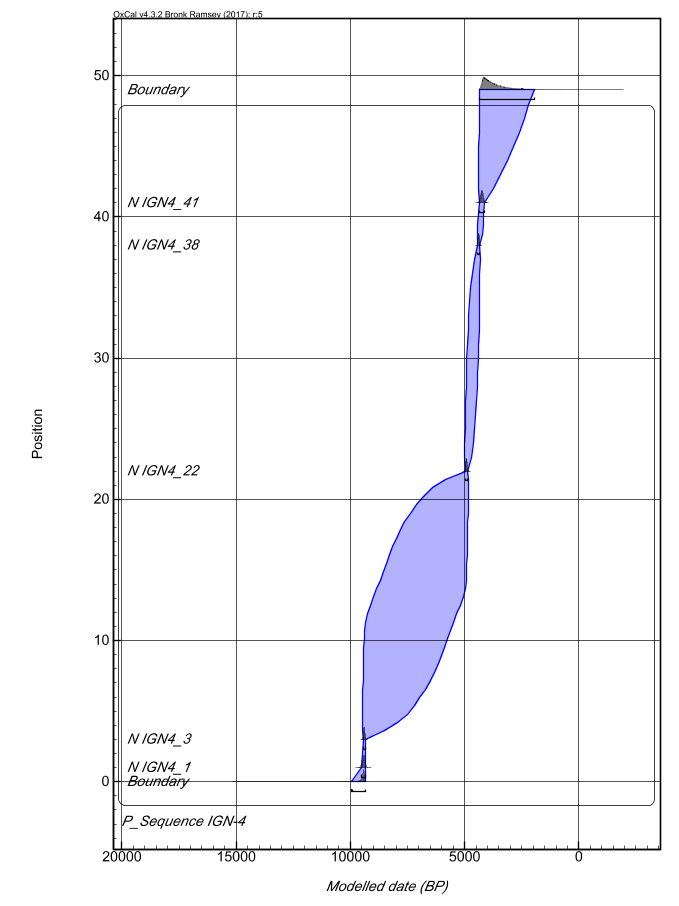


Fig. S3-4. Growth model for sample IGN4 (stalagmite). IGN4_1 through IGN4_41 – measured ages. Grey areas depict 0.945 probability distribution at fixed positions. Blue area depicts 0.945 probability boundaries.

## Flowstone IGN5


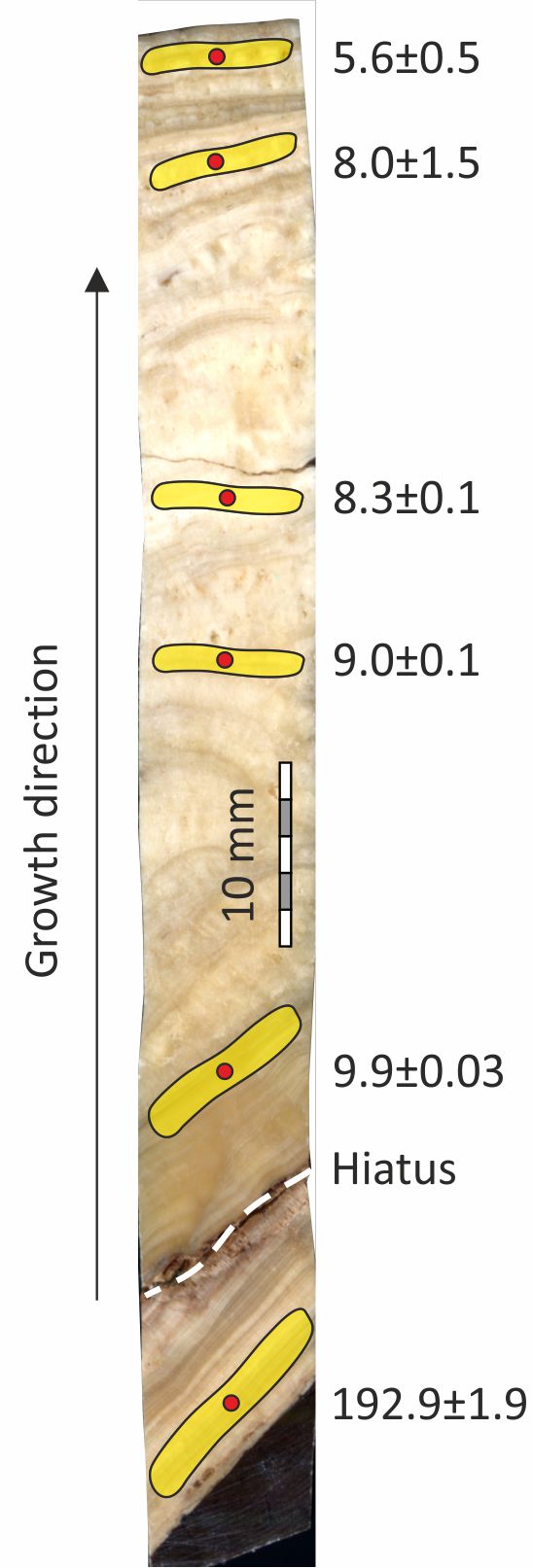


Fig. S3-5. Sample IGN5. Outlines of layers taken for dating are shown. Red dots indicate positions used to define distances from base of the crust. Numbers are ^230^Th ages ±2σ uncertainty (ka BP).


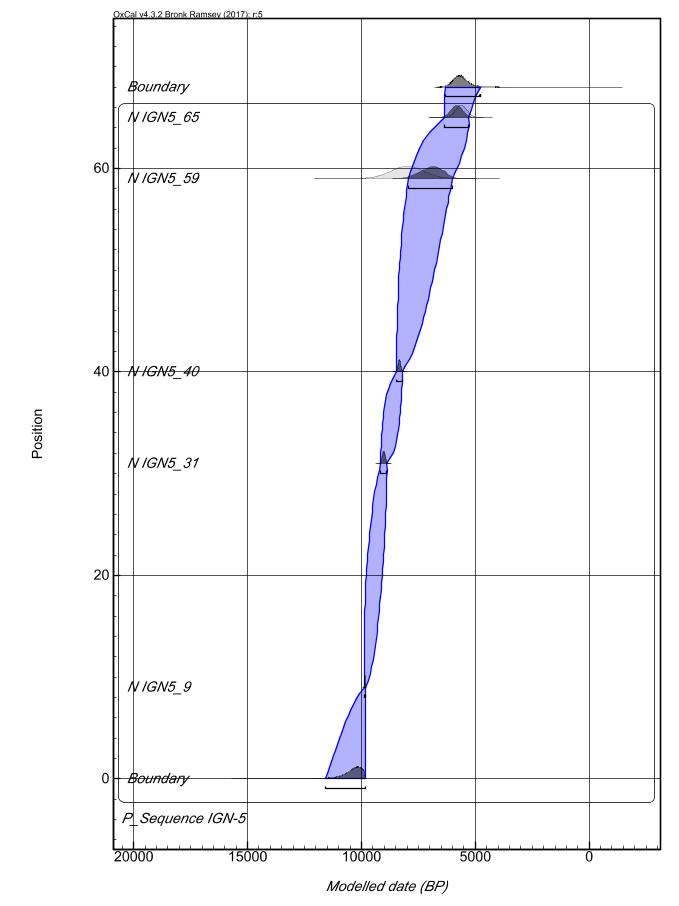


Fig. S3-6. Growth model for sample IGN5. IGN5_9 through IGN5_65 – measured ages. Grey areas depict 0.945 probability distribution at fixed positions. Blue area depicts 0.945 probability boundaries.

## Stalagmite IGN


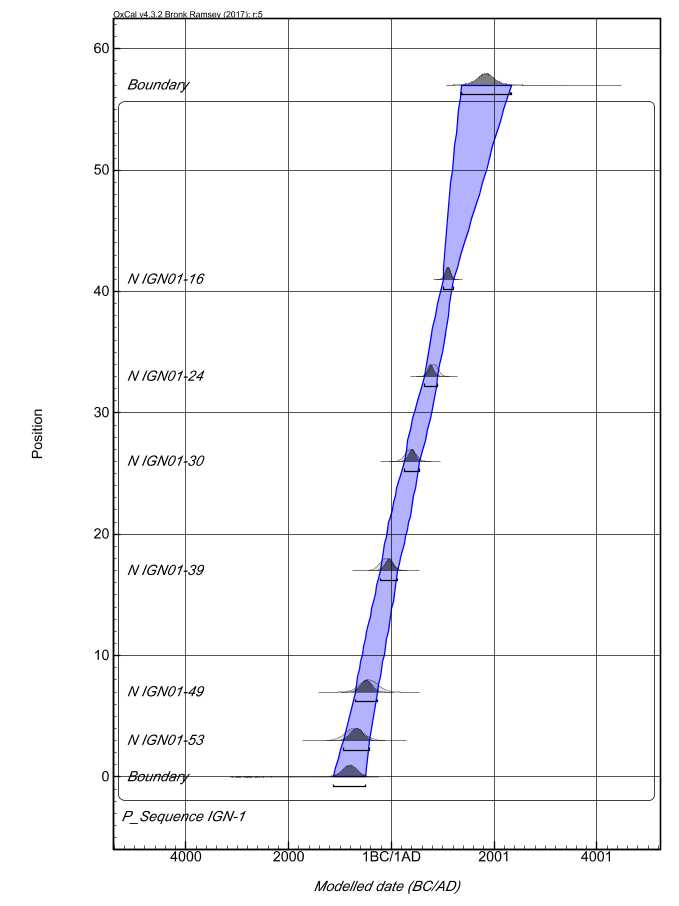


Fig. S3-8. Growth model for stalagmite IGN IGN1_16 through IGN1_53 – measured ages. Grey areas depict 0.945 probability distribution at fixed positions. Blue area depicts 0.945 probability boundaries.


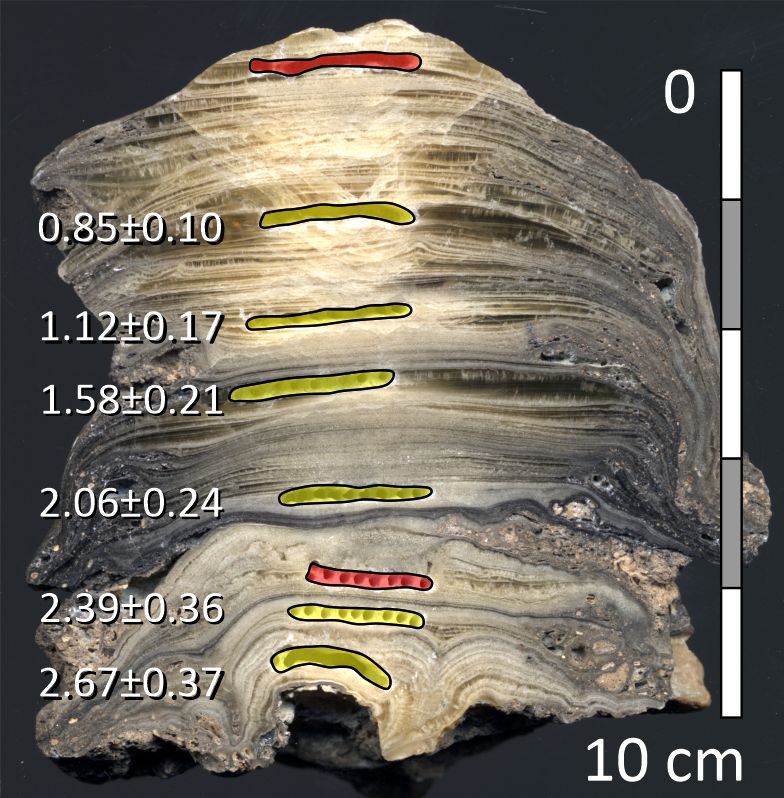


Fig. S3-7. Stalagmite IGN with ^230^Th sub-sample positions indicated (red indicates failed analyses). Numbers are ^230^Th ages ±2σ uncertainty (ka BP).

# Section S4. Summary of ^14^C dates from the cultural layer of Ignatievskaya cave

Table S4

| Spl ID | Mate-rial | ^14^C date  (BP) | Calibrated age (a BP) | | | | | | Notes, source |
| --- | --- | --- | --- | --- | --- | --- | --- | --- | --- |
|  |  |  | @ 1σ | | Probability | @ 2σ | | Probability |  |
|  |  |  | min | max |  | min | max |  |  |
| Pit II | | | | | | | | | |
| IEMEA-366 | B | 14,038±490 | 16,352 | 17,662 | 0.682 | 15,752 | 18,329 | 0.954 | Layer 2а, upper part (0.05−0.25 m). Composite bone sample. “Visiting layer”, enriched in charcoal and cultural remains. Smirnov *et al*. (1990). |
| IPAE-41† | B | 13,500 ±1,660 | 13,984 | 18,484 | 0.682 | 12,634 | 21,827 | 0.954 | Layer 2а, lower part (0.45−0.55 m). Bone remains indistinguishable from those from upper part of the layer (sample IEMEA-366). Smirnov *et al*. (1990). |
| SBAS-2468‡ | C | 10,400±465 | 11,599 | 12,710 | 0.634 | 11,060 | 13,189 | 0.926 | Layer 3.2 (0.03−0.15 m). Petrin (1992). |
| SBAS-2209 | C | 14,240±150 | 17,121 | 17,542 | 0.682 | 16,887 | 17,776 | 0.954 | Layer 3.2 (0.05−0.25 m). Petrin (1992). |
| OxA-39967 | C | 13,892±39 | 16,826 | 17,002 | 0.683 | 16,697 | 17,035 | 0.954 | Layer 0.02−0.06 m. Above dark layer. Dublyansky *et al*. (2020). |
| OxA-39968 | C | 13,605±37 | 16,345 | 16,499 | 0.683 | 16,289 | 16,580 | 0.954 | Layer 0.06−0.07 m. Upper dark layer. Dublyansky *et al*. (2020). |
| OxA-39969 | C | 13,936±38 | 16,912 | 17,037 | 0.683 | 16,773 | 17,071 | 0.954 | Layer 0.07−0.08 m. Lens-shaped layer between two dark layers. Dublyansky *et al*. (2020). |
| OxA-39970 | C | 14,232±39 | 17,272 | 17,391 | 0.481 | 17,105 | 17,413 | 0.954 | Layer 0.08−0.09 m. Lower dark layer. Dublyansky *et al*. (2020). |
|  |  |  | 17,147 | 17,217 | 0.201 |  |  |  |  |
| OxA-39971 | C | 23,878±93 | 27,810 | 28,085 | 0.683 | 27,760 | 28,296 | 0.954 | Layer 0.12−0.18 m. Below lower dark layer. Dublyansky *et al*. (2020). |

Table S4 (Continued)

| Spl ID | Mate-rial | ^14^C date  (BP) | | Calibrated age (a BP) | | | | | | Notes, source |
| --- | --- | --- | --- | --- | --- | --- | --- | --- | --- | --- |
|  |  |  |  | @ 1σ | | Probability | @ 2σ | | Probability |  |
| UBA-42155§ | B | 21,052±91 | | 25,396 | 25,566 | 0.389 | 25,175 | 25,660 | 0.954 | Layer 0.06−0.07 m. Upper dark layer. Fragments of teeth (marmot and hare). Dublyansky *et al*. (2020). |
|  |  |  |  | 25,263 | 25,389 | 0.294 |  |  |  |  |
| UBA-42156§ | B | 20,542±84 | | 24,610 | 24,920 | 0.683 | 24,357 | 25,024 | 0.954 | Layer 0.08−0.09 m. Lower dark layer. Five bone fragments (pika and hare or marmot). Dublyansky *et al*. (2020). |
| Pit III | | | | | | | | | | |
| IEMEA-365 | C | 13,335±192 | | 15,755 | 16,302 | 0.682 | 15,416 | 16,616 | 0.954 | Layer 3.2 (0.05−0.25 m). Petrin (1992). |
|  |  |  |  |  |  |  |  |  |  |  |

*Notes*: Spl ID – sample identifier. Material: C – charcoal, B – bone. OxA and UBA dates obtained by AMS; all other analyses – by radiometric method. Calibration performed using OxCal 4.4 (Bronk Ramsey 2008; Bronk Ramsey & Lee, 2013) and IntCal20.f14c (Reimer *et al*. 2020). † – questionable date; ‡ – date labeled as possibly erroneous in the original publication (Petrin 1992: 163); § – old ages due to sediment turbation (Dublyansky *et al*., 2020). Positions of arheological pits are shown in Fig. 2 in the main paper.

*Laboratory codes*: IPAE – Institute of Plant and Animal Ecology, Ural Branch, Russian Academy of Sciences, Yekaterinburg, Russian Federation; IEMEA – Institute of Evolutional Morphology and Ecology of Animals, Russian Academy of Sciences, Moscow, Russian Federation; SBAS –Institute of Geology and Mineralogy, Siberian Branch, Russian Academy of Sciences, Novosibirsk, Russian Federation; OxA – Oxford Radiocarbon Accelerator Unit, Oxford, UK; UBA – 14CHRONO Centre, Belfast, Northern Ireland.

In this table the original Cyrillic laboratory codes are converted to their Latin equivalents using English versions of the official names of organizations. Note that three radiocarbon dates shown in the table were reported in Steelman *et al*. (2002) under different laboratory codes: SBAS (Siberian Branch of Academy of Sciences) as LSBRAS (Laboratory of Siberian Branch of Russian Academy of Sciences) and IEMEA (Institute of Evolutional Morphology and Ecology of Animals) as Institute of Evolutional Morphology and Ecology of Animals (unabbreviated).

# Section S5. ^14^C dates of black paintings of Ignatievskaya cave

Table S5

| Spl ID | ^14^C date  (BP) | Calibrated age (a BP) | | | | | | Painting |
| --- | --- | --- | --- | --- | --- | --- | --- | --- |
|  |  | @ 1σ | | Probability | @ 2σ | | Probability |  |
|  |  | min | max |  | min | max |  |  |
| CAMS-56586 | 7,370±50 | 8,237 | 8,309 | 0.275 | 8,044 | 8,325 | 0.954 | Mammoth |
|  |  | 8,158 | 8,221 | 0.324 |  |  |  |  |
|  |  | 8,061 | 8,088 | 0.083 |  |  |  |  |
| CAMS-56271 | 7,920±60 | 8,918 | 8,970 | 0.120 | 8,602 | 8,982 | 0.954 | Radial line |
|  |  | 8,831 | 8,863 | 0.084 |  |  |  |  |
|  |  | 8,633 | 8,785 | 0.464 |  |  |  |  |
| CAMS-67688 | 6,030±110 | 6,736 | 7,011 | 0.658 | 6,637 | 7,175 | 0.954 | Line |

*Notes*: Original data were reported by Steelman et al. (2002). All analyses were made by AMS. Spl ID – sample identifier. Dates were re-calibrated using OxCal 4.4 (Bronk Ramsey 2008; Bronk Ramsey & Lee, 2013) and IntCal20.f14c (Reimer et al. 2020).

# References

Bronk Ramsey, C. (2008). Deposition models for chronological records. *Quaternary Science Reviews*, 27, 42–60. doi:10.1016/j.quascirev.2007.01.019

Bronk Ramsey, C., Higham, T., Bowles, A., & Hedges, R. (2007). Improvements to the pretreatment of bone at Oxford. *Radiocarbon*, 46, 155–163. doi:10.1017/S0033822200039473.

Bronk Ramsey, C., & Lee, S. (2013). Recent and planned developments of the program OxCal. *Radiocarbon*, 55, 720–730. doi:10.1017/S0033822200057878

Cheng, H., Edwards, R.L., Shen, C-C., Polyak, V.J., Asmerom, Y., Woodhead, J., … Alexander Jr, E.C. (2013). Improvements in ^230^Th dating, ^230^Th and ^234^U half-life values, and U–Th isotopic measurements by multi-collector inductively coupled plasma mass spectrometry, *Earth and Planetary Science Letters*, 371–372, 82–91. doi:10.1016/j.epsl.2013.04.006.

Dublyansky, Y., Kosintsev, P., Shirokov, V., Spötl, C. (2020). Posescheniya Ignatievskoy peschery lyudmi v pozdnem paleolite: utocjneniye i rashireniye radiouglerodnoy khronologii. *Rossiiskaia arkheologiia*. Submitted http://ras.jes.su/ra/s086960630012516-6-1.

Edwards, R.L., Chen, J.H., & Wasserburg, G.J. (1987). ^238^U, ^234^U, ^230^Th, ^232^Th systematics and the precise measurement of time over the past 500,000 years. *Earth and Planetary Science Letters,* 81, 175−192. doi:10.1016/0012-821X(87)90154-3

Jaffey, A. H., Flynn, K. F., Glendenin, L. E., Bentley, W. C., & Essling, A. M. (1971). Precision measurement of half-lives and specific activities of ^235^U and ^238^U. *Physical Review* *C,* 4, 1889−1906. http://doi.org/10.1103/PhysRevC.4.1889

Petrin, V.T. (1992). *Paleoliticheskoe sviatilishche v Ignatievskoi peshchere na Yuzhnom Urale* [Paleolithic sanctuary in Ignatievskaya cave in Southern Ural]. Novosibirsk, Nauka.

Reimer, P., Austin, W., Bard, E., Bayliss, A., Blackwell, P., Bronk Ramsey, C., … Talamo, S. 2020. The IntCal20 Northern Hemisphere radiocarbon age calibration curve (0–55 cal kBP). *Radiocarbon*, 62, 725–757. doi:10.1017/RDC.2020.41

Shen, C.-C., Wu, C.-C., Cheng, H., Edwards, R. L., Hsieh, Y.-T., Gallet, S., … Spötl, C. (2012). High-precision and high-resolution carbonate ^230^Th dating by MC-ICP-MS with SEM protocols. *Geochimica et Cosmochimica Acta*, 99, 71–86. doi:10.1016/j.gca.2012.09.018

Smirnov, N.G., Bolshakov, V.N., Kosintsev, P.A., Panova, N.K., Korobeynikov, Y.I., Olshvang, V.N., … Bykova, G.V. (1990). *Istoricheskaya ekologiya zhivotnykh gor Yuzhnogo Urala* [Historical ecology of animals of the Southern Ural Mountains]. Sverdlovsk, UrO AN SSSR.

Steelman, K., Rowe, M., Shirokov, V., & Southon, J. (2002). Radiocarbon dates for pictographs in Ignatievskaya Cave, Russia: Holocene age for supposed Pleistocene fauna. *Antiquity,* 76, 341−348. doi:10.1017/S0003598X00090426

van Klinken, G.J. (1999). Bone collagen quality indicators for palaeodietary and radiocarbon measurements. *Journal of Archaeological Science*, 26, 687–695. [doi:10.1006/jasc.1998.0385](https://doi.org/10.1006/jasc.1998.0385)

Wedepohl, K. H. (1995). The composition of the continental crust. *Geochimica et Cosmochimica Acta,* 59, 1217−1232. doi:10.1016/0016-7037(95)00038-2
